# Monitoring by Parents and Hypothesized Male-Female Differences in Evidence from a Nationally Representative Cohort Re-sampled from Age 12 to 17 Years: An Exploratory Study Using a “Mutoscope” Approach

**DOI:** 10.1007/s11121-014-0517-8

**Published:** 2014-11-29

**Authors:** Ryan B. Seedall, James C. Anthony

**Affiliations:** 1 Department of Family, Consumer, and Human Development, Utah State University, Logan, UT USA; 2 Department of Epidemiology & Biostatistics, Michigan State University College of Human Medicine, 909 Fee Road, East Lansing, MI USA

**Keywords:** Parental monitoring, Male-female differences, Limiting time with friends

## Abstract

The link between adept parental monitoring (PM) and later positive behavioral and health outcomes already has motivated intervention trials, but questions remain about which specific facets and mechanisms of PM make a difference. Our current research questions concern fundamental male-female differences in PM facets as manifest in a US cohort, re-sampled each year at age 12 through 17 years during an interval from 2004 to 2009. We hypothesized emergence, by mid-adolescence, of a specific male-female difference in a “limit time with friends” (LTF) facet of adept PM, with overall PM levels held constant. The data, arranged using a “mutoscope” approach, are from six successive nationally representative independent cross-sectional sample surveys of the cohort, with each adolescent measured only once, via a multi-item PM module nested within the larger survey. Estimates and tests of male-female differences are from a “multiple indicators, multiple causes” latent structure model appropriate for complex survey data. In evidence consistent with the advance hypothesis and with PM level held constant via the model, the LTF facet generally was more relaxed for boys as compared to girls, in a difference that emerged by mid-adolescence, possibly due to greater LTF constraints for girls at mid-adolescence. This research adds to the knowledge base about male-female similarities and differences in facets of PM. As a specific PM facet, LTF might function as a mechanism suitable for deliberate intervention and as a possible specific target in “micro-trials” of new prevention research. We acknowledge limitations such as omitted variables, including social media effects, not measured in this investigation’s national surveys, but of potential importance in future research on peer influence as might have more distal parenting determinants.

## Introduction

Most prevention researchers already are quite aware of the importance of adept monitoring of children and adolescents by their parents, with parental monitoring (PM) defined to encompass supervision of peer relations and placing limits on time with friends. Adept PM has been linked to a variety of positive behavioral and health outcomes (e.g., see Dishion and Tipsord 2011). Specific potential benefits of adept monitoring include lower levels of affiliation with rule-breaking peers (e.g., see Dishion et al. 1991; Lloyd and Anthony 2003), as well as lower incidence rates and risks of becoming a newly incident user of alcohol, tobacco, and other drugs (ATOD; Chilcoat et al. 1995; Chilcoat and Anthony 1996; Cohen et al. 1994), less frequent ATOD use (Steinberg et al. 1994), and a lower likelihood of progression to heavier drinking or other drug use (Reifman et al. 1998; Reboussin and Anthony 2001).

This well-substantiated knowledge base about adept monitoring and its positive outcomes already has become a foundation for randomized clinical intervention and prevention trials intended to strengthen families through parental education. The resulting evidence from these trials, to date, often is positive and supportive. Some prevention trials indicate that intervention fidelity and implementation science issues are crucial. For example, after informed consent processes are used to recruit families, the volunteering parent participants can be encouraged but not required to attend the intervention sessions. In this manner, assessment of intervention effects can be fragile when parents fail to attend the sessions as planned. Nonetheless, even with unfaithful attendance, positive outcomes have been found (e.g., see Dishion and Andrews 1995; Ialongo et al. 1999; Storr et al. 2002; Dishion and Tipsord 2011; Spoth et al. 2011). Outcomes to date also note the importance of research on variability of monitoring behaviors and their effectiveness. More evidence is needed to make intervention and prevention efforts more adaptive and customizable to individualized or subgroup-specific needs of families.

### Adolescent Age and Sex Differences in Parental Monitoring

Adolescent age and sex differences are seen for PM overall, and in estimates of PM influence on health behaviors. In several studies, older adolescents typically are supervised less closely and have more experiences outside the family structure (Patterson and Stouthamer-Loeber 1984; Larson et al. 1996), concurrent with increased adolescent autonomy and bids for independence (Steinberg 1990), as well as affiliation with norm-violating peers (e.g., see Lloyd and Anthony 2003). Monitoring older adolescents can also be more challenging. This fact might help to increase salience of adolescent disclosure regarding “where they are going, what they will be doing, and whom they will be with” (Kerr et al. 2010, p. 39).

Evidence for variable PM trajectories is clear. In some studies, there is a steady decrease in monitoring over time for both boys and girls (Li et al. 2000; Spano et al. 2011). Other studies show relative stability in PM levels during middle-late childhood and early adolescent years (e.g., see Patterson and Stouthamer-Loeber 1984; Larson et al. 1996; Reboussin and Anthony 2001; Lloyd and Anthony 2003). Even so, Dishion et al. (2004) draw attention to a possibility that precocious child or adolescent independence or autonomy might be a marker of excess risk of negative behavioral outcomes and can disrupt otherwise adept monitoring.

Perhaps with PM levels or facets as causal determinants, male-female differences also can be seen in various domains of adolescent externalizing behavior, conduct problems, and other hazard-laden experiences, with greater occurrence among males for some of these behaviors and experiences (e.g., marijuana smoking, getting into physical fights, and not wearing a seat belt in a car driven by someone else), and with greater occurrence among females for other behaviors and experiences (e.g., forced to have sexual intercourse, and riding with a driver who had been drinking alcohol; Eaton et al. 2006). Nonetheless, in terms of parental monitoring generally, previous findings indicate that PM levels for girls generally are higher than PM levels for boys in childhood and during adolescence (e.g., see Bumpus et al. 2001; Reboussin and Anthony 2001; Webb et al. 2002; Svenson 2003; Lloyd and Anthony 2003). Variations in monitoring of boys versus girls might be related to a male-female double standard in terms of societal influences, attitudes, and expectations related to courtship and family formation (Axinn et al. 2011). The Axinn research team offers empirical evidence of a parental “tendency to hold significantly more permissive attitudes toward a future son’s courtship experiences than a future daughter’s courtship experiences” (p. 429).

### Investigating Specific Facets of Parental Monitoring

PM has multiple facets, some of which might be anticipated to exert differential effects on adolescent behaviors, with potential implications for prevention science, along the lines of mechanism-informing substudies and micro-trials advocated by Howe et al. (2010). These discrete facets include specific parent behaviors (e.g., limiting behaviors and soliciting information, and encouraging adolescent disclosure), each of which might serve as indicators of monitoring, or that might have effects with PM levels held constant. In a recent calibration investigation intended to stimulate new prevention research, we investigated the possibility that drug prevention effects of specific parenting practices, in the specific form of setting limits on time with friends, might be large enough to have clinical or public health significance. We chose the “limit time with friends” (LTF) facet of PM because it represents an important indicator of parental behavior designed to monitor and possibly influence peer relationships and prevent “hanging out with the wrong crowd”—that is, LTF might be used to control or regulate otherwise unregulated and generally ubiquitous peer influences. Implications for prevention of health-related behaviors such as extra-medical use of internationally regulated drugs become clear when the issue of limiting time with friends surfaces. One especially robust predictor of adolescent drug involvement is whether adolescents have friends who are also drug users, and the LTF facet of parenting might have a direct influence on affiliation with drug-using or otherwise deviant peers (e.g., see Dishion et al. 2004). In theory, the LTF facet of parenting can be quite malleable and amenable to change via brief interventions with parents—perhaps more readily changed than other PM facets, particularly if an intervention session is followed by “boosters” as might be provided via automated SMS text messaging to a parent’s mobile phone, a topic to which we will return in this paper’s Discussion section.

That is, as an attempt to substantiate the potential impact and importance of research on the LTF facet of parenting, our calibration investigation sought to learn whether LTF-associated differences in estimated incidence of adolescent drug use might approach the size of observed male-female incidence differences—with knowledge that these male-female differences are considered to be of clinical and public health significance, sufficient to foster a National Institute on Drug Abuse initiative on sex and gender differences in drug taking. In our already published work on LTF, we found that the estimated LTF effect on incidence of drug might be as large as or larger than recently observed male-female differences in risk of becoming a drug user (Seedall and Anthony 2013).

### Purpose of This Study

Building upon these recent findings, we set out to probe the LTF facet of PM in an effort to build a stronger empirical foundation for potential future use in a targeted micro-trial or other trials of interventions designed to enhance PM. With respect to LTF, we expected no male-female difference in the level of the LTF facet of parenting in early adolescence, but we thought that the LTF disparity would arise during the middle years of adolescence, with LTF levels for females becoming larger than those for males in later adolescence, when courtship and unwanted pregnancies become more salient as parenting concerns in relation to daughters relative to sons (Axinn et al. 2011).

In addition, the PM knowledge base requires renewal from time to time due to possible secular or cohort changes in parenting practices. As a result, we designed this study to provide an initial exploration, via a novel “mutoscope” approach as described below, of twenty-first century patterns of the overall PM construct while also working toward our primary aim of seeking information regarding potential age and male-female differences in the intensity of the LTF facet of monitoring and supervision.

**Fig. 1:**
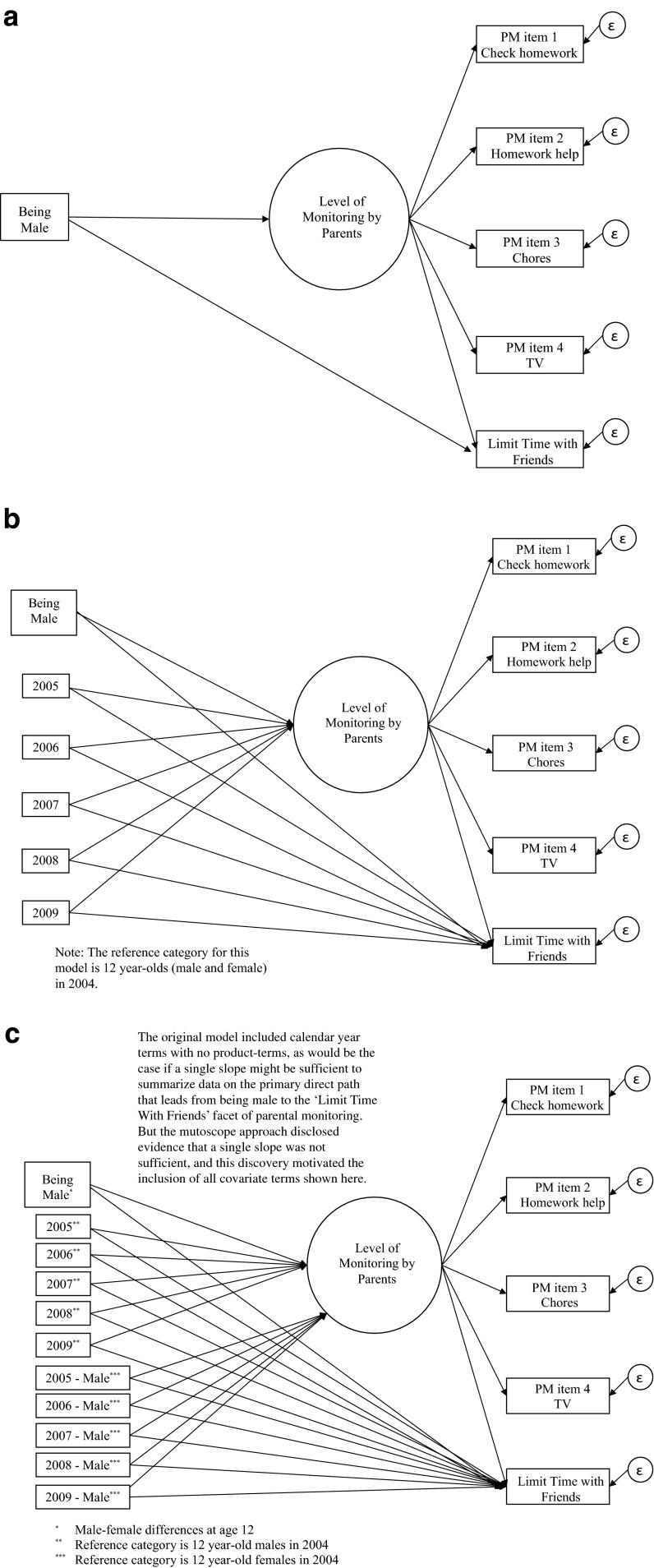
**a** Model to depict hypothesized male-female differences in ‘limit time with friends’ with overall parental monitoring level held constant under the model. **b** Model to depict hypothesized male-female differences in ‘limit time with friends’ with overall parental monitoring level and age subgroup membership held constant under the model. **c** Model to depict hypothesized male-female differences in ‘limit time with friends’ with overall parental monitoring level held constant under the model, with an allowance for male-female differences to vary across age subgroups

This report describes some *a priori * expectations with respect to our specific interest in the LTF facet of parenting, with overall PM levels as a background issue. As already mentioned, we thought that there might be no LTF differences in male-female contrasts as childhood ends and adolescence begins (i.e., at age 12 years), with overall PM levels held constant. Thinking developmentally, we expected to see emergence of a specific male-female LTF difference once issues of worry about precocious sexual activity and pregnancy became more salient during the adolescent years, especially by age 17 years, still with overall PM levels held constant. Conceptual models depicting the main steps in our analysis plan are displayed in Fig. 1a–c.

An important methodological limitation of this exploratory study, to which we will return in later sections of the paper, involves measurement equivalence and assumptions that have to be made about the metric of the overall PM latent variable. Under the models fit here, for our primary hypothesis about the LTF facet of the PM construct, we use the standard regression approach to hold constant overall PM levels while estimating this male-female difference in LTF values as observed. Because we have 12 subgroups under study (boys and girls in each of six age-year substrata), we have not tried to establish PM measurement equivalence as might allow us to draw more definitive conclusions about how the PM levels vary across these subgroups, which would require much larger samples of each age-sex subgroup than are now available. We can offer preliminary estimates for male-female and age differences based on an untested assumption about PM measurement equivalence, but our primary focus is on estimation of male-female variation in the LTF facet, which does not require measurement equivalence for the overall PM construct. Statistically informed readers may appreciate the context in relation to the concept of statistical power to detect differences. Once we subdivide these samples into age-sex strata, the number of individual participants in each subgroup stratum becomes so small that there is a quite constrained statistical power to detect a departure from measurement equivalence. Instead, we estimate the possibility of an LTF difference emerging as this nationally representative sample of a birth cohort of males and females ages from 12 to 17 years, with PM level held constant, and we leave the issue of male-female differences in PM level as a topic for future and more definitive research to confirm PM measurement equivalence across the age-sex strata under study.

## Materials and Methods

### Study Population, Sampling, and Assessment Procedures

The 2004–2009 National Surveys on Drug Use and Health (NSDUH) represent examples of sustained epidemiological surveillance, with non-overlapping samples drawn each year from national probability sampling frames designed to include non-institutionalized US citizens, age 12 years and older. The NSDUH research team takes special care to ensure nationally representativeness of the samples via multi-stage area probability sampling and recruitment protocols. NSDUH participants complete a multi-module audio-enhanced computer-assisted self-interview (ACASI) for gathering of information on hundreds of variables relating to a variety of health-related issues, including alcohol and drug use, mental health, and general health concerns. From 2004 to 2009, the NSDUH total sample consisted of 333,732 respondents, with just under one third of respondents being between the ages of 12 and 17 years (*n* = 108,560).

Our specific sample was generated from a longitudinal trace consisting of 18,551 respondents, as distributed in the year-age strata shown in Table 1. That is, a nationally representative US sample of 12-year-olds was drawn, recruited, and assessed with the ACASI modules in 2004. A new non-overlapping sample of that same original cohort was drawn, recruited, and assessed in exactly the same manner, each year from 2005 to 2009, such that the cohort of 12-year-olds in 2004 had become 13 years old in 2005, 14 years old in 2006, and so on. The intent is for no individual to be assessed more than once (i.e., sampling without replacement). Arising from successive nationally representative sample surveys, the large numbers of boys and girls at each age enhance statistically precise and generalizable study estimates.

**Table 1:** Description of the epidemiological sample under study, starting with males and females age 12 years in 2004, and the successively re-sampled cohort, year by year, at each successive age

Age		Year 2004	2005	2006	2007	2008	2009
12	M F *n*	1,441 1,433 2,874					
13	M F *n*		1,567 1,544 3,111				
14	M F *n*			1,614 1,475 3,089			
15	M F *n*				1,594 1,485 3,079		
16	M F *n*					1,638 1,583 3,221	
17	M F *n*						1,592 1,585 3,177

Data from the US National Survey on Drug Use and Health (NSDUH), 2004–2009 (aggregate *n* = 18,551)

To conceptualize this research approach, consider an analogy using inventor Herman Casler’s early twentieth century mutoscope, which involved taking a series of single assessment cross-sectional snapshots of an object in motion, arranging the snapshots on cards, and then flipping the photos in succession so that the cascading imagery of cross-sectional snapshots might be converted into a primitive movie of the object in motion (http://www.flipbook.info/viewers.php, last accessed 23 October 2014). Here, our mutoscope approach involves arranging each set of data from each re-sampling of the nationally representative cohort in relation to strata or subgroups defined by the age at assessment, the year of assessment, and sex (male vs female). The 12-year-old boys in the nationally representative sample of 2004 form one stratum and the 12-year-old girls of 2004 form another stratum, allowing direct comparison of PM practices, such as the LTF facet. Then, the 13-year-old boys sampled and assessed for the first time in 2005 provide a snapshot view of PM practices when that same cohort had passed the 13th birthday, and the 13-year-old girls sampled and assessed for the first time in 2005 provide a snapshot view of PM when they had passed the 13th birthday (i.e., with a new sample of cohort members). The remaining strata of boys and girls are formed in a similar fashion, age by age, and year by year, through to 2009, when the national cohort of 12-year-olds in 2004 reach age 17 years.

This mutoscope approach to repeated “panel study” data yields a trace of cross-sectional single assessment snapshots of a cohort’s experience from one age to the next, with each member of the sample assessed once and only once. As such, this research approach to understanding PM stability and change reduces all sample attrition problems faced when trying to track down and re-assess a cohort sampled just one time, and also brings potential measurement reactivity and interdependence to a minimum (see Anthony 2010). This approach constrains interdependence of observations in repeated measures research—i.e., interdependence such that an individual’s measured values of PM at time *t + 1* might be found to depend upon his or her measured values of PM at a prior time *t*. Overall, this approach creates a novel way to test developmental hypotheses about male-female differences in PM practices as the cohort makes transitions into and through the adolescent years, somewhat conceptually similar to but fundamentally different from an accelerated longitudinal cohort study design.

### Measures

The multi-module ACASI assessment included five different items pertinent to adept monitoring and supervision by parents, each of which was included in the PM construct used in this study as a discrete categorical indicator variable with four-point Likert scale levels from 1 (never) to 4 (always). These items tapped how often in the past year parents had limited time with friends (*M* = 2.94; *SD* = 1.08), limited time watching television (*M* = 2.07; *SD* = 1.06), checked homework (*M* = 3.20; *SD* = 0.95), helped with homework (*M* = 3.28; *SD* = 0.99), or assigned work or chores (*M* = 3.33; *SD* = 0.78). A particular strength of these items is that they are all malleable and modifiable aspects of parenting, at least theoretically amenable to parent education and interventions.

### Analysis Plan

We chose to integrate the description of our analysis/estimation steps with presentation of results in order to describe the steps and their results with greater clarity. The analysis/estimation strategy was preceded by Tukey-style exploratory data analysis (EDA) to illuminate marginal distributions of these study variables. Model-fitting steps are depicted in cartoon-like Fig. 1a–c, as described in the Results section. Anonymous reviewers and the journal’s methods editorial staff suggested some of the post-estimation exploratory data analysis steps described below. All analysis/estimation steps were completed using MPlus software, Version 7, with an approach designed to be appropriate for analysis-weighted complex survey data and “subpopulation” specifications.

## Results

### Analytic Strategy

Our plan for data analysis was organized in relation to a standard multi-step approach that concluded with a multiple indicators, multiple causes (MIMIC) model within an exploratory structural equation modeling (ESEM) framework using MPlus, after EDA. As outlined recently by Asparouhov and Muthen (2009), ESEM allows for greater model flexibility than confirmatory factor analysis (CFA) while it also includes more pre-defined information than exploratory factor analysis (EFA). In addition, although the MIMIC model has been most often used within item response theory to detect item bias in the form of differential item functioning (DIF), we used it in the context of this study to test our specific substantive hypotheses about selective male-female LTF differences (see Gallo et al. 1994; Edelen et al. 2009). Use of MIMIC modeling within an ESEM framework made it possible for us to explore associations between age, sex, and PM, and also to shed light on inter-relationships linking age, sex, and LTF while holding constant levels of the general PM construct, as described in our introductory overview of the research approach. In these analyses, the metric of the latent PM construct is uncertain, and we do not claim that the metric for boys is exactly the same as the metric for girls. In this initial exploratory study with the mutoscope approach, we decided not to confirm measurement equivalence for the 12 subgroups under study (boys vs girls across each of six age-year strata), as would be required to draw more definitive inferences about levels of the latent PM construct. For this reason, our description of estimates gives emphasis to the slope estimates for the paths leading directly toward the LTF indicator, which is anchored in the observed item response categories from 1 to 4 (i.e., not necessarily ordinal, but at least ordered category values).

### Preliminary Modeling Step 1: Male-Female Differences Generally

In an initial estimation step, we fit to the data a parsimonious regression model for complex survey sample data that is based on the possibly over-simplified assumptions that age and year make no difference. The resulting “common regression slope” estimate of the male-female difference in PM levels borrows information across all years and age groups. We then elaborated that model to allow for the possibility of a male-female difference in the LTF facet of monitoring, also summarized in the form of a common regression slope based on all ages and all years, and on the over-simplified assumptions. Figure 1a shows the conceptual model with the direct effect path from the sex variable to the LTF indicator, as well as the indirect path via the PM construct.

The parsimonious regression model used to estimate male-female differences across all years and age groups was based upon an assumption that PM and LTF might not vary for males and females across year or age group. This model fits the data no better than moderately well, *χ* ^2^ (8, *n* = 18,511) = 810.7, *p* < 0.001; CFI = 0.88; RMSEA = 0.07 (90 % confidence interval (CI) = 0.07; 0.08). A table is not needed to summarize the two slope estimates from this model. The primary estimate disclosed similarly robust male-female differences in relation to the LTF variable (estimated slope, *b* = −0.145; standard error, SE = 0.023; Taylor series *p* < 0.001). Parents seem to be more lax in their limits set for boys’ time with friends, with PM level held constant under this model. The exploratory slope estimate of secondary importance indexes possible male-female differences in PM level, has a positive sign, and is statistically robust (*b* = 0.06; SE = 0.03; *p* = 0.04; Table 2). Subject to an untested assumption of measurement equivalence, this secondary slope suggests that parents might be monitoring boys at higher levels than girls across all age cohorts. We will return to this interesting topic in our Discussion section.

**Table 2:**
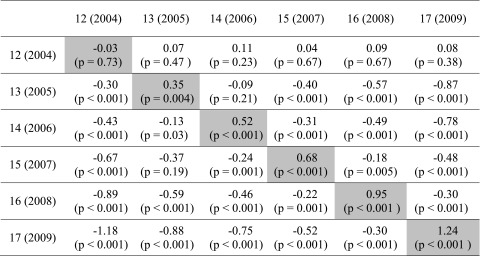
Estimated differences in the overall level of parental monitoring (PM), contrasting males versus females (highlighted diagonal trace), within-male age differences (upper right cells), and within-female age differences (lower left cells)

Data from the United States National Survey on Drug Use and Health (NSDUH), 2004–2009 (*n* = 18,551). Main Diagonal Trace—statistically robust (*p* < 0.05) higher PM levels for boys versus girls, age by age, with age 12 years as an exception (no appreciable male-female difference in PM at age 12 years). Upper right cells—row-wise age contrast for boys at age *x* + 1 versus boys at age *x.* Lower left cells—row-wise age contrast for girls at age *x* + 1 versus girls at age *x*

### Preliminary Modeling Step 2: Age Differences

In the next estimation step, we posited a model that was a more realistic portrayal of our expectations for age-related variations, allowing for the possibility that an age adjustment might lead to a change in the estimated direct path being used to estimate male-female differences in the LTF facet of PM. This modeling goal was accomplished by adding dummy-coded variables that represent the cohort as sampled at each age (Fig. 1b). For example, the first age variable in the series (a4) coded 12-year-old respondents in 2004 as 1 with all others coded 0, the second age variable (a5) coded 13-year-old respondents in 2005 as 1 with all others coded 0, and so on.

With the model re-specified with these age terms, the fit statistics improved somewhat, χ^2^ (23, *n* = 18,511) = 1121.8, *p* < 0.001; CFI = 0.81; RMSEA = 0.05 (90 % CI = 0.048; 0.053). With PM level held constant under this model, the slope to estimate male-female differences in LTF did not change appreciably from the *b* = −0.145 value found in step 1 (step 2, *b* = −0.15; *p* < 0.001; data not shown in a table). There was an incrementally positive slope for LTF from the 12-year-old cohort sample onward. LTF slope estimates for age cohorts 13–17 were observed as tangibly larger than values observed for the 12-year-old cohort, but Wald tests of parameter constraints designed to test for differences revealed that the incremental differences age by age were not appreciably different from one another relative to conventional standards (i.e., all *p* > 0.05 for age 13 vs 14, 14 vs 15, etc.).

With respect to our exploratory estimation of slopes leading toward the latent PM construct, there was an inverse slope estimate year by year, when looking across the samples of the cohort, from 12-year-olds sampled in 2004 to counterparts in the sample of 17-year-olds in 2009. Subject to our untested assumption of measurement equivalence, the inverse slopes indicate that PM level for 12-year-olds in 2004 was higher than the PM level for 13-year-olds in 2005. Also subject to this assumption, an inverse slope estimate also was seen across the span of time as the 12-year-old cohort observed in 2004 moved forward across later age and time strata (data not shown in a table).

### Primary Estimation Step: Variations Across Sex-Age Subgroups

In this primary estimation step, we posited a model that more completely expressed our expectation that with overall PM level held constant via the regression model, the parents might relax LTF differentially for boys as we look across the strata from the 12-year-olds in 2004 to the 17-year olds in 2009, but this might not be the case for girls. Here, the approach involved forming dummy-coded variables with product terms for the sex-age subgroups from age 13 to age 17 (Fig. 1c). The resulting model yielded the following model fit statistics: χ^2^ (38, *n* = 18,511) = 637.0, *p* < 0.001; CFI = 0.83; RMSEA = 0.03 (90 % CI = 0.027; 0.031).

Most central to our primary hypothesis about male-female differences in LTF values under this model, there was no male-female difference in the 2004 contrast of boys and girls age 12 years (*b* = −0.01, SE = 0.06, *p* = 0.89, upper left cell of Table 3). Even so, as can be seen by following the diagonal trace downward and to the right, the hypothesized male-female difference in the LTF facet of parenting was seen to emerge and to be statistically robust in the other contrasts (i.e., in mid-later adolescence). For each age cohort across the adolescent age range from 13 to 17 years, boys’ time with friends was restricted at lower levels as compared to girls of the same age (*p* < 0.05; Table 3 diagonal trace). These results serve as evidentiary support in favor of our primary LTF-related hypothesis that there might be no difference in LTF for pre-teen boys versus girls but that LTF differences might be substantial for the 16- and 17-year-olds in this cohort, with PM level held constant under the model. We also note a similar male-female difference in LTF for the 13–15-year-olds in this cohort.

**Table 3:**
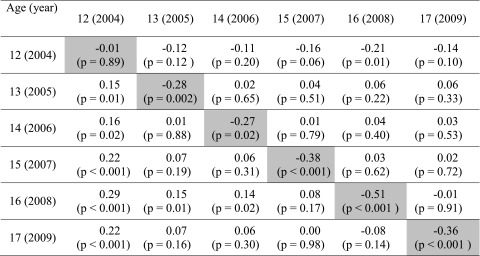
Estimated differences in the limiting time with friends (LTF) facet of parental monitoring, contrasting males versus females (highlighted diagonal trace), within-male age differences (upper right cells), and within-female age differences (lower left cells), under the multivariate response model that holds constant overall level of parental monitoring

Data from the United States National Survey on Drug Use and Health (NSDUH), 2004–2009 (*n* = 18,551). Trace—statistically robust (*p* < 0.05) lower LTF levels for boys versus girls, age by age, with age 12 years as an exception (no appreciable male-female difference in LTF at age 12 years). Upper right cells—row-wise age contrast for boys at age *x* + 1 versus boys at age *x*. Lower left cells—row-wise age contrast for girls at age *x* + 1 versus girls at age *x*

Subject to the untested measurement equivalence assumption, we looked for evidence of systematic variation in overall PM levels. Under this model, there was no statistically robust male-female difference in PM level in early adolescence at age 12 years in 2004 (*b* = −0.03; SE = 0.07; *p* = 0.73). Nonetheless, a statistically robust male-female difference emerged in each subsequent year, but with an unexpected outcome. Namely, if indeed the measurement metric is equivalent, the observed overall parental monitoring levels seem to be higher for boys than for girls from ages 13 to 17 years (see Table 2, diagonal trace).

If one were to assume PM measurement equivalence across all 12 subgroups, the evidence shown in Table 2 (above diagonal trace) would be consistent with generally hypothesized expectations about age and PM levels. Estimates in the above diagonal (upper right) cells of Table 2 are for boys. These exploratory estimates suggest an age-associated decline in overall PM levels for boys contrasted from one age to the next, beginning with contrasts between 13- and 15-year-olds (*b* = −0.40; *p* < 0.001) and continuing to contrasts between 16- and 17-year-olds (*b* = −0.30; *p* < 0.001). PM levels for boys might have increased slightly between the ages of 12 and 13 years before beginning to decrease. Estimates for girls, as shown in the lower left below diagonal cells of Table 2, disclose a roughly similar pattern. To illustrate (again with an assumption of measurement equivalence across subgroups), the girls at age 12 years in 2004 were found to have higher PM levels under this model as compared to girls at each older age. Table 2 (lower left below diagonal) shows evidence suggesting PM levels at statistically significant lower values for girls across the independent samples drawn 1 year after the other (e.g., *b* = −0.30 for girls in 2005 vs girls in 2004, *p* < 0.001; *b* = −1.18 for girls in 2009 vs girls in 2004, *p* < 0.001). If confirmed in future research, these findings would tend to support maturational model, possibly with general PM construct levels decreasing over time for boys and girls.

## Discussion

### Brief Summary of Findings

In this study, we investigated hypothesized age-specific male-female differences in the limit time with friends facet of parental monitoring with PM held constant (as measured by the LTF item as well as items on limiting time watching television, checking homework, helping with homework, and assigning work or chores). Our main hypothesis concerned the LTF facet of the PM construct, and the findings confirm our initial expectation of male-female LTF differences, with exploration of possible male-female and age-related variations in PM levels.

The general picture is one of clear male-female differences in the LTF facet of parenting of adolescent daughters versus sons in recent years within the USA. The successive cross-sectional snapshots from early-late adolescence showed evidence of more permissive parenting with respect to allowing sons to spend time with friends as compared to daughters spending time with friends, but not at age 12 years, in models holding constant overall PM levels age by age. This evidence, coupled with findings from our previous study that LTF risk differences for newly incident drug use were at least as large as male-female differences in newly incident drug use, may help support and provide a rationale for a specific focus on the LTF facet in parenting interventions and in future drug use prevention trials (Seedall and Anthony, 2013).

In our more exploratory findings on overall PM levels, we did not place emphasis on the possibility of male-female differences, due to unresolved measurement equivalence issues. The evidence is consistent with developmental or maturation-related variations such that the levels of overall monitoring might generally decrease across successive ages, for both males and females in adolescence, but more work on the overall PM measurement equivalence assumption is needed before firm conclusions should be drawn.

### Limitations

Before a more detailed discussion of these results and their implications, several of the more important of this study’s limitations merit attention. One limitation worth noting is the study’s cross-sectional sample in each year, which provides a population-averaged snapshot but not a subject-specific view of an individual’s change over time. It has been necessary to use the population-averaged mutoscope approach and to yoke the successive snapshots in sequence in order to gain a longitudinal view of the 12-year-old cohort sampled and measured in 2004, as that cohort matured out to age 17 years.

In relation to limitations, we also are reminded of an issue raised by Gault-Sherman (2012)—namely, the bidirectionality of parent–offspring relationships that cannot be fully illuminated in any cross-sectional research project. Nonetheless, here, in this study, we are quite confident that PM and LTF values have not altered the values of the male-female sex variable, nor the age variable.

Against this background information on limitations, we note that a counterbalanced strength is in these annual surveys that seek nationally representative samples of each age cohort under study in the USA. As a result, although the 12-year-olds in the 2004 survey are never re-sampled or re-assessed in successive years, each year’s sample is nationally representative of the nation’s children and adolescents, year by year. Via inspection of year-to-year progress of each age cohort, it is possible to surmount disadvantages faced in longitudinal research, including differentially selective attrition, as well as potential reactivity in assessments, both of which sometimes limit interpretation in longitudinal samples (Anthony 2010). Of course, despite strengths of this type, we acknowledge that evidence from longitudinal research projects provides more information about within-individual differences, which always will serve as useful complements to epidemiological investigations of this type.

We also would like to highlight two other issues related to the research approach used in the NSDUH to assess PM. One aspect of research approach involves an interpretation of what is being measured. From one perspective, the important constructs under study are adept parental monitoring and supervision, and limit setting about time with friends, which are being measured by what the sons and daughters report. From another perspective, we can view the boys’ and girls’ responses to the survey assessments as a measure of what they perceive about how and how well they are being monitored by their parents (e.g., see Kerr et al. 2010; Stattin and Kerr 2000).

That is, in this formulation, the construct might be said to be “perceived level of parental monitoring” or “perceived LTF,” as opposed to actual “levels of PM and LTF.” We surmise that each reader of this research report might choose to adopt one perspective versus another, with corresponding constraints on the interpretation of the findings, and with allowance for the possibility that it is the reporting of PM and LTF that varies from one age to the next, as opposed to our own interpretation that it is the actual PM and LTF values that vary from one age to the next. Deliberately designed experiments will be required to tease apart whether the important variables are “what is perceived,” versus “what is disclosed,” versus “what actually was experienced.”

We also have noted that all of the five PM items used in the NSDUH assessment represent parental behaviors than can be readily modified, and this is an important feature of the NSDUH assessment from the standpoint of future public health interventions. Nonetheless, it also is important to acknowledge that these five items do not necessarily capture all facets of adept parental monitoring. In this regard, we concur that PM might be cast in future research as a multi-dimensional construct based upon mutually influencing parent and child behaviors, with an allowance that a PM level as perceived and reported by a child (or by a parent) might be different from a PM level measured via an externally verifiable measurement device (Kerr et al. 2010). Whereas the present nationally representative NSDUH sample survey has remarkable strengths in relation to external generalizability and the sample space, a research project focused more specifically on parenting of adolescents almost certainly would have a more comprehensive PM and LTF assessment, making it possible to probe the PM and LTF issues in more depth than has been possible in this nationally representative sample survey context. We acknowledge that the overall PM levels for girls might be found to be greater than the overall PM levels for boys, if the NSDUH had employed a more comprehensive PM survey module, and if measurement equivalence can be substantiated.

### Overall Implications

Notwithstanding limitations such as these, the study findings are of interest for a variety of reasons, including external generalizability of the evidence, traced back to a nationally representative study sample of adolescents in the community, as well as the strengths of a standardized assessment plan across the multiple survey years and the size of the samples under study. As for implications, we have chosen to concentrate our attention on the possibility that the LTF facet of parental monitoring might deserve more careful attention in clinical and public health population–oriented outcomes research, perhaps with greater attention to the LTF facet specifically within the context of preventive interventions that seek to improve overall PM in a more general way. We speculate that the LTF facet might be a crucial component of PM and parental supervision in general, with an important functional interdependence—i.e., interdependent with and perhaps determinative of the age at which sons and daughters are first exposed to a chance to try alcohol or tobacco or to experiment with cannabis, opioids, or other internationally regulated drugs. This is a research question to which our attention now will turn, given supportive evidence of LTF variations by sex and by age, as discovered in this new look at parental monitoring and supervision of adolescents.

It is in the context of tobacco and other drug prevention research that the value of an LTF focus might be seen. To illustrate, we hope that trialists will consider our previously mentioned “micro-trials” approach to assess whether PM intervention impact on these drug outcomes might be strengthened when a random subset of participating parents is chosen for SMS text messaging or other interventions specifically directed to the LTF facet. That is, consistent with a micro-trials probe into the potential mechanisms of intervention effect, there could be an overall randomization to the PM intervention (e.g., parent education sessions and DVDs) and to the control condition, and then a more individualized SMS messaging about LTF might be assigned at random to those receiving the PM intervention. In this way, the specific utility of LTF might be seen in trial outcomes such as a delayed first chance to smoke tobacco or try other drugs, reduced levels of affiliation with drug-using peers, and delayed or reduced onset of underage smoking or other precocious drug use, as gauged against any potentially salubrious background effects of an intervention directed toward increasing the more general PM levels.

## Conclusion

Overall, this study sheds light on potentially important male-female differences in parenting behaviors that might foster new and more probing research into the LTF facet of monitoring and supervision during late childhood and early adolescence. Our findings provide additional evidence for what Axinn et al. (2011) call a double standard, with girls being limited more on issues relating to courtship or family formation. It was beyond the scope of our current study to address whether these male-female differences are associated with more positive or negative outcomes (such as involvement with alcohol, tobacco, or the internationally regulated drugs). Nonetheless, we are hopeful that the results from this study will foster new research on the potential public health significance of the observed variations, including prevention trials with interventions that have a strengthened focus upon specific PM facets, such as limiting time with friends. Through new prevention experiments of this type, it should become possible to estimate the degree to which it is LTF per se that conveys the effect of parenting interventions, or whether these effects are mediated entirely by what the adolescent is willing to disclose to parents, or what the adolescent perceives about parental monitoring. In this way, the prevention research on parenting can be used to shed light on fundamental issues of adolescent development, including the male-female differences observed here.
